# Reexpression of *LSAMP* inhibits tumor growth in a preclinical osteosarcoma model

**DOI:** 10.1186/1476-4598-13-93

**Published:** 2014-04-28

**Authors:** Tale Barøy, Stine H Kresse, Magne Skårn, Marianne Stabell, Russell Castro, Silje Lauvrak, Antonio Llombart-Bosch, Ola Myklebost, Leonardo A Meza-Zepeda

**Affiliations:** 1 Department of Tumor Biology, Institute for Cancer Research, Oslo University Hospital, The Norwegian Radium Hospital, Oslo, Norway; 2 Department of Pathology, Medical School of Valencia University, Valencia, Spain; 3 Department of Molecular Biosciences, University of Oslo, Oslo, Norway; 4 Genomics Core Facility, Oslo University Hospital, Oslo, Norway

**Keywords:** Osteosarcoma, *LSAMP*, Tumor suppressor, *LSAMP-AS1*, *LSAMP-AS3*, *LSAMP-AS4*, Deletion 3q13.31, Proliferation, Tumor formation

## Abstract

**Background:**

Osteosarcomas are the most common primary malignant tumors of bone, showing complex chromosomal rearrangements with multiple gains and losses. A frequent deletion within the chromosomal region 3q13.31 has been identified by us and others, and is mainly reported to be present in osteosarcomas. The purpose of the study was to further characterize the frequency and the extent of the deletion in an extended panel of osteosarcoma samples, and the expression level of the affected genes within the region. We have identified *LSAMP* as the target gene for the deletion, and have studied the functional implications of *LSAMP-*reexpression*.*

**Methods:**

*LSAMP* copy number, expression level and protein level were investigated by quantitative PCR and western blotting in an osteosarcoma panel. The expression of *LSAMP* was restored in an osteosarcoma cell line, and differences in proliferation rate, tumor formation, gene expression, migration rate, differentiation capabilities, cell cycle distribution and apoptosis were investigated by metabolic dyes, tumor formation *in vivo,* gene expression profiling, time-lapse photography, differentiation techniques and flow cytometry, respectively.

**Results:**

We found reduced copy number of *LSAMP* in 45/76 osteosarcoma samples, reduced expression level in 25/42 samples and protein expression in 9/42 samples. By restoring the expression of *LSAMP* in a cell line with a homozygous deletion of the gene, the proliferation rate *in vitro* was significantly reduced and tumor growth *in vivo* was significantly delayed. In response to reexpression of *LSAMP,* mRNA expression profiling revealed consistent upregulation of the genes hairy and enhancer of split 1 (*HES1)*, cancer/testis antigen 2 (*CTAG2)* and kruppel-like factor 10 *(KLF10).*

**Conclusions:**

The high frequency and the specificity of the deletion indicate that it is important for the development of osteosarcomas. The deletion targets the tumor suppressor *LSAMP,* and based on the functional evidence, the tumor suppressor function of *LSAMP* is most likely exerted by reducing the proliferation rate of the tumor cells, possibly by indirectly upregulating one or more of the genes *HES1*, *CTAG2* or *KLF10*. To our knowledge, this study describes novel functions of *LSAMP*, a first step to understanding the functional role of this specific deletion in osteosarcomas*.*

## Background

Osteosarcomas are the most common primary malignant tumors of bone. They are highly aggressive with poor prognosis [1,2] and occur most frequently in children and adolescents [3]. The efficacy of the current treatments has reached a plateau, and the need of increased biological understanding is crucial to improve treatment options and thus the life of patients.

At the genomic level, osteosarcomas show complex chromosomal rearrangements with multiple gains and losses [4,5]. Array comparative genomic hybridization (aCGH) has been used extensively to analyze DNA copy number changes at a higher resolution, identifying recurrent chromosomal alterations [6-11]. We have previously identified a novel, frequent deletion in 3q13.31 in osteosarcomas [6]. Of the genes located within the deleted region, three have been proposed to be involved in cancer biology: the protein-coding gene limbic system-associated membrane protein (*LSAMP*) and the two non-coding RNAs LSAMP RNA antisense 3 (*LSAMP-AS3*) (also known as *LOC285194* or *TUSC7*) and LSAMP RNA antisense 4 (*LSAMP-AS4*) (also known as *BC040587*) [6,12-20]. *LSAMP* has previously been reported to be a candidate tumor suppressor gene in clear cell renal cell carcinoma and epithelial ovarian cancer [15-17], and subsequently also in osteosarcomas [6,12-14]. All three of these genes have also been proposed to act in conjunction as tumor suppressors in osteosarcomas [13].

In this study, the frequency and extent of the deletion and the aberrations of *LSAMP* were further investigated. To study the potential importance of *LSAMP* in osteosarcoma biology, we have examined the functional implications of *LSAMP-*reexpression in an osteosarcoma cell line with a homozygous deletion of the gene.

## Results

### The deletion in 3q13.31 targets *LSAMP*

**Figure 1:**
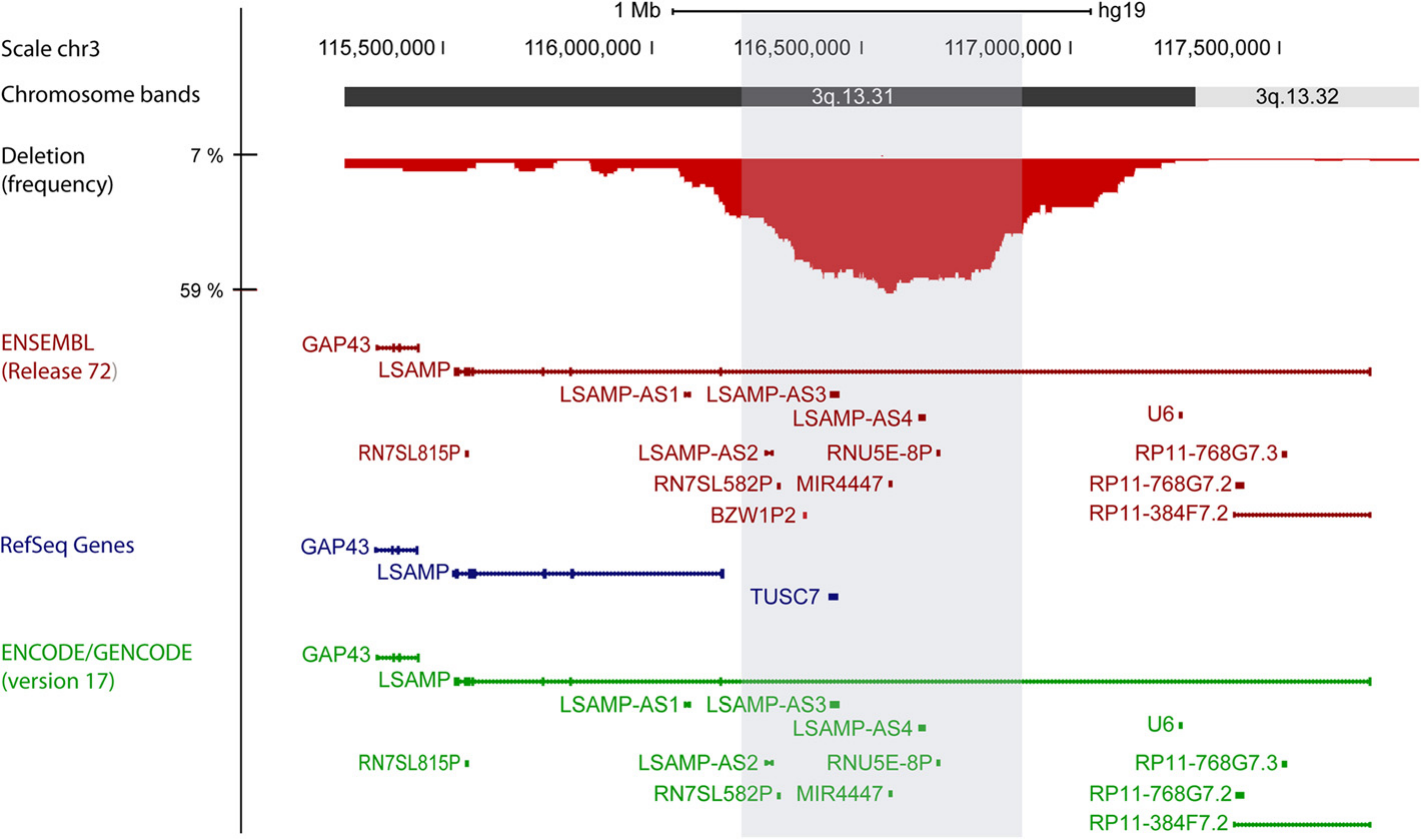
**Chromosome map and frequency plot of the observed deletions in 3q13.31 in osteosarcoma samples (n = 76).** The shaded gray area (chr3:116,269,000-116,896,000) corresponds to a frequency of ≥ 30% of the samples. Below the frequency plot are the genes within 3q13.31 annotated by the databases ENSEMBL (red) and RefSeq (blue), and supporting annotation by ENCODE/GENCODE data (green).

In order to precisely define the deletion in 3q13.31, high-resolution DNA copy number data obtained using Affymetrix Genome-Wide Human SNP Array 6.0 on a total of 76 osteosarcoma samples (32 clinical samples, 25 xenograft samples and 19 cell lines) ( [21,22] and Kresse *et al*., unpublished) were investigated. We determined the minimal recurrent deletion to be from chr3:116,560,000-116,577,000 and present in 59% (45/76) of the samples (Figure 1), with a similar distribution across the different sample types (56% of the clinical samples (18/32), 64% of the xenograft samples (16/25) and 58% of the cell lines (11/19)). No differences were observed among the different osteosarcoma subtypes investigated, although the majority of the samples were of osteoblastic subtype (subtype information in Additional file 1: Table S1). The high frequency suggests that loss of 3q13.31 is important for development of osteosarcoma, and that the region may harbor tumor suppressor gene(s).

The number of annotated genes within the deleted region differs between the two databases ENSEMBL and RefSeq, with the newly published ENCODE/GENCODE data (version 17) supporting the ENSEMBL annotation (Figure 1). Within the deleted region (chr3:116,000,000-117,500,000), there are two genes commonly annotated by both databases; *LSAMP* and *LSAMP-*AS3 (or *TUSC7)* (Figure 1). To investigate whether loss of other genes besides *LSAMP* could be important, we performed gene expression analysis of LSAMP RNA antisense 1 (*LSAMP-AS1*), *LSAMP-AS3* and *LSAMP-AS4* in a panel of 5 osteosarcoma clinical samples, 13 xenograft samples, 19 cell lines and 14 control samples (n = 51). Expression of *LSAMP-AS1* was detected in 30/51 samples, with a similar level between the osteosarcoma samples and the control samples (Additional file 2: Figure S1). Furthermore, since *LSAMP-AS1* is located in the flanking region of the deletion (Figure 1), it was excluded from further experiments. Low expression of *LSAMP-AS3* was detected in 5/46 samples, independent of the DNA copy number status, but not in any of the control samples (Additional file 2: Figure S1). Expression of *LSAMP-AS4* was not detected in any of the samples, cancer nor control (0/42) (Additional file 2: Figure S1). In comparison, expression of *LSAMP* was detected, although in variable amounts, in 43/49 of these samples, including all the control samples ( [6] and Barøy *et al.*, unpublished). These results indicate that the deletion in 3q13.31 is not inactivating any of the genes *LSAMP-AS1, LSAMP-AS3* or *LSAMP-AS4,* but rather that the expression level, or lack thereof, is a normal state for both non-cancerous and cancerous cells. Thus, *LSAMP* is most likely the target gene for the deletion.

### Aberrations of *LSAMP*

**Figure 2:**
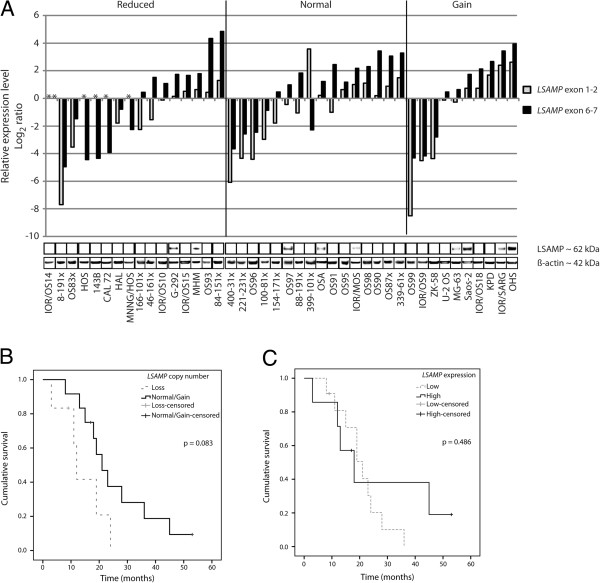
**LSAMP aberrations and patient survival. A**: The correlation between *LSAMP* copy number, expression level and protein level in osteosarcoma samples (n = 42). The samples are sorted according to their copy number (reduced, normal or gain). The relative expression level was measured by qRT-PCR using two probes to cover the length of the gene (located in exon junction 1–2 and 6–7). No detectable expression level is indicated with an asterisk (*). The corresponding LSAMP protein level was determined using western blot, with β-actin as loading control. **B**: Kaplan-Meier plot showing overall survival for patients with loss (n = 6) and normal/gain (n = 12) of *LSAMP* copy number. **C**: Kaplan-Meier plot showing overall survival for patients with low (n = 11) and high (n = 7) expression of *LSAMP,* compared to the average expression of two normal bone samples.

Aberrations of *LSAMP* were investigated at the copy number, expression and protein level (Figure 2A) in 42 osteosarcoma samples (8 clinical samples, 13 xenografts and 21 cell lines). The copy number and expression level of all the cell lines, except CAL 72 and G-292, have been determined previously using aCGH and qRT-PCR, respectively [6]. The copy number and expression levels of the remaining samples were determined using TaqMan DNA Copy Number Assay and qRT-PCR, respectively. In total, 16/42 (38%) of the samples had reduced copy number, 16/42 (38%) had normal copy number and 10/42 (24%) had increased copy number. There were no differences between sample types or osteosarcoma subtypes. Of the 16 samples with loss of copy number, 11 samples had no or lower expression of *LSAMP* compared to the average expression level of two normal bone samples, detected by at least one of the probes (Figure 2A). Of the 16 samples with normal copy number, 9 samples had lower expression of *LSAMP* detected by at least one of the probes. Of the 10 samples with increased copy number, 5 samples had lower expression of *LSAMP* detected by at least one of the probes, indicating that copy number aberrations might not be the only mechanism regulating the expression level of *LSAMP.* In total, 25/42 (60%) samples showed reduced expression level compared to the normal bone samples.

The protein level was investigated by western blotting (Figure 2A). Of the samples with loss of copy number, 2/16 had detectable levels of the LSAMP protein, shown by a band of approximately 62 kDa, corresponding to the size reported by others (60–68 kDa) [23,24]. Of the samples with normal copy number, 3/16 had detectable levels of the LSAMP protein, whereas of the samples with gain of copy number, the protein was detected in 4/10 samples. In total, the protein was detected in one clinical sample and eight cell lines. There was no clear correlation between mRNA level and protein level, as some samples with relatively high mRNA level had undetectable protein levels.

We have previously shown an association between low expression of *LSAMP* and poor survival [6]. Of the samples investigated in this study (n = 18, of which 10 xenografts and 8 clinical samples), although not statistically significant (*p* = 0.083, Mantel-Cox test), there was a trend towards poorer survival in patients with loss of *LSAMP* copy number (Figure 2B). There was no association between the expression of *LSAMP* and overall survival (Figure 2C) (*p* = 0.486).

### Restoring the expression of *LSAMP*

**Figure 3:**
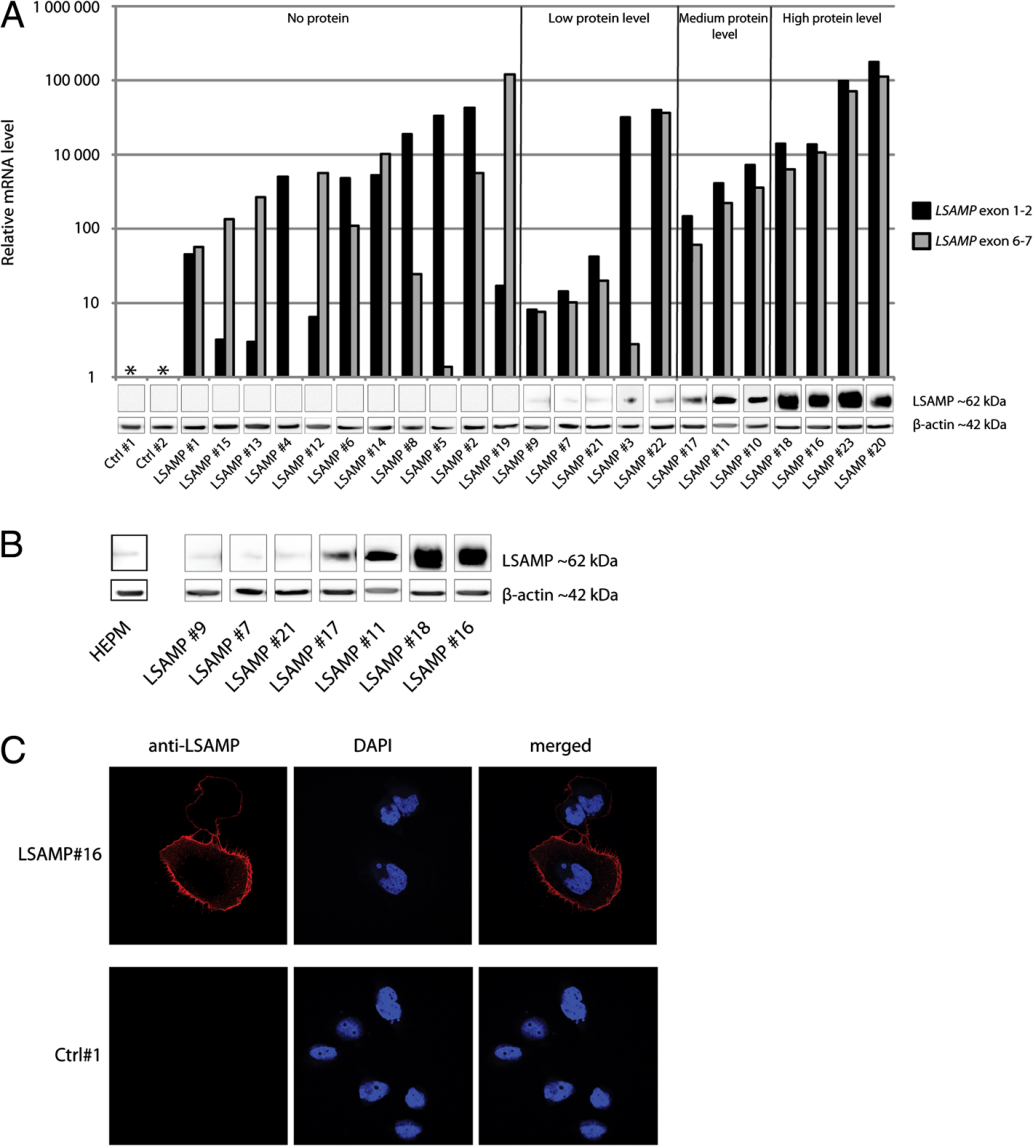
**LSAMP reexpression. A**: *LSAMP* expression and protein level in clones with ectopic expression of *LSAMP*. The expression level was measured using qRT-PCR, using two probes (located in exon junction 1–2 and 6–7). The corresponding LSAMP protein level was determined using western blot, with β-actin as loading control. The clones are sorted according to whether they have undetectable, low, medium or high protein levels. **B**: Comparison of the endogenous levels of the LSAMP protein in the non-cancerous cell line HEPM and seven *LSAMP*-expressing clones with increasing amount of protein. **C**: Subcellular location of the ectopically expressed LSAMP protein in *LSAMP*-expressing cells (LSAMP #16) and control cells (Ctrl #1) determined using immunofluorescence confocal microscopy. Red color represents stain for anti-LSAMP-antibody, blue represents staining of the nuclei (DAPI).

The cell line IOR/OS14 was chosen to ectopically reexpress *LSAMP* as it has a homozygous deletion of the gene [6]. In total, 23 clones transfected with *LSAMP* ORF were assayed for levels of ectopic *LSAMP* mRNA and protein, and compared to two control clones (backbone vector). All 23 clones had detectable levels of the *LSAMP* mRNA, but only 12 showed detectable though variable protein levels (Figure 3A). The clones were categorized to whether they had undetectable, low, medium or high levels of the LSAMP protein (Figure 3A). Low protein levels were most comparable to the endogenous protein levels found in osteosarcoma samples investigated (Figure 2A) and the non-cancerous mesenchymal cell line HEPM (Figure 3B). By immunofluorescence confocal microscopy, the ectopically expressed LSAMP protein was shown to be localized to the cell membrane (Figure 3C), which is consistent with LSAMP being a membrane protein [25].

### Ectopic expression of *LSAMP* delays tumor formation *in vivo*

**Figure 4:**
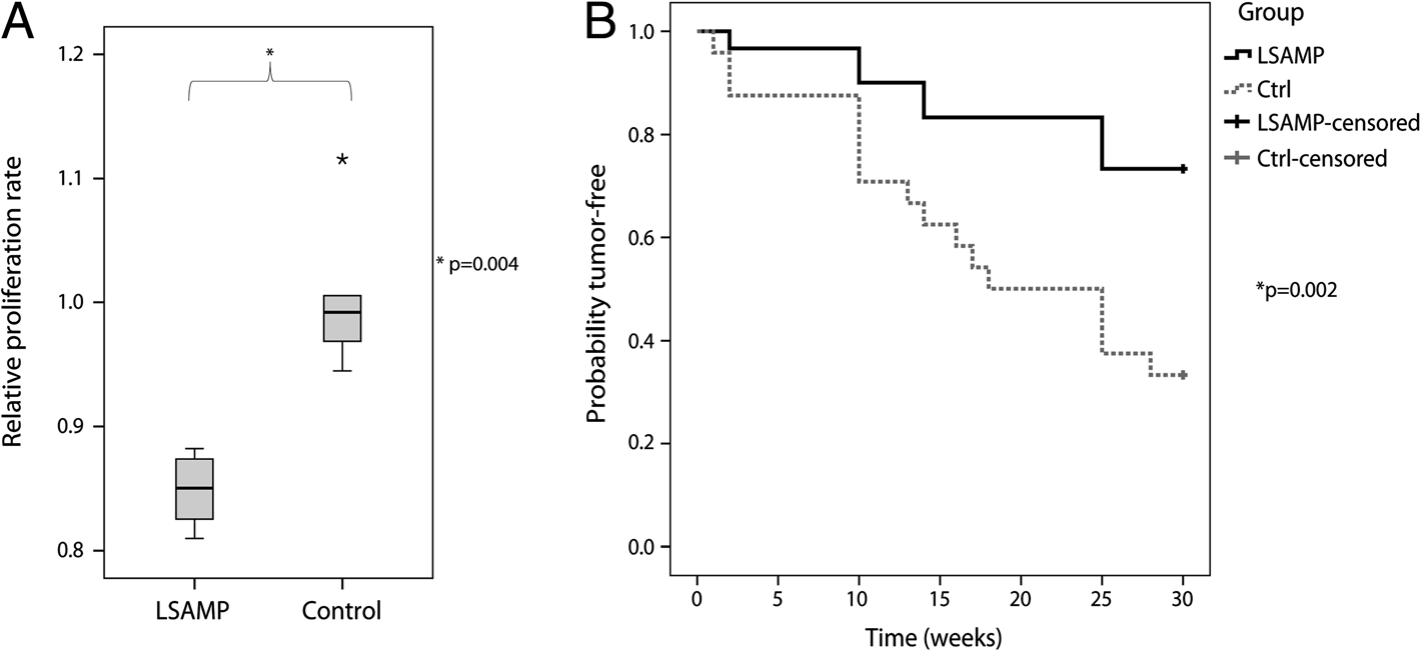
**Proliferation rate and *in vivo *tumor growth. A**: Relative proliferation rate of *LSAMP-*expressing cells (LSAMP #7, #9 and #21) compared to cells without *LSAMP-*expression (Ctrl #1 and #2 and non-transfected cells). The experiment was performed twice. The midline is the median observation, and the whiskers represent the total spread of the observations. The difference was tested statistically significant with a Mann–Whitney test (*p* = 0.004). **B**: *In vivo* tumorigenicity measured as time until tumor appearance, represented by a Kaplan-Meier plot. The difference between the *LSAMP-*expressing cells (LSAMP #7, #9 and #21) and the cells without *LSAMP-*expression (Ctrl #1 and #2) was tested statistically significant by a Mantel-Cox test (*p* = 0.002).

The clones with low levels of the LSAMP protein (LSAMP #7, #9 and #21) were chosen for functional characterization and compared to cells without *LSAMP-*expression (Ctrl #1, #2 and non-transfected cells). The proliferation rate of the clones with the *LSAMP* protein was significantly reduced 15-20% compared to the cells without *LSAMP*-expression (Figure 4A) (*p* = 0.004, Mann–Whitney test).

With exception of non-transfected cells, the same clones were used for investigating the *in vivo* tumor-forming ability of the cells*.* Each clone was injected into both flanks of six mice, giving a total of 12 potential tumor sites, with the exception of LSAMP #9, which was injected into three mice and thus had a total of six potential tumor sites. Time until tumor appearance is shown by a Kaplan-Meier plot in Figure 4B, showing that cells with the *LSAMP* protein have a significant delayed tumor formation compared to the clones without *LSAMP*-expression (*p* = 0.002, Mantel-Cox test).

In addition, other cancer phenotypes were investigated. Neither cell cycle distribution (Additional file 3: Figure S2) nor apoptosis (Additional file 4: Figure S3) identified by flow cytometry, or the migration rate using time-lapse photography in the IncuCyte (Additional file 5: Figure S4), were shown to be affected by the expression of *LSAMP*. As the parental cell line IOR/OS14 has been shown to successfully differentiate towards the adipogenic and osteogenic lineage [26], differences in differentiation capabilities were investigated. The degree of differentiation was not affected by the expression of *LSAMP* (data not shown).

### Ectopic expression of *LSAMP* upregulates *HES1*, *CTAG2* and *KLF10*

**Figure 5:**
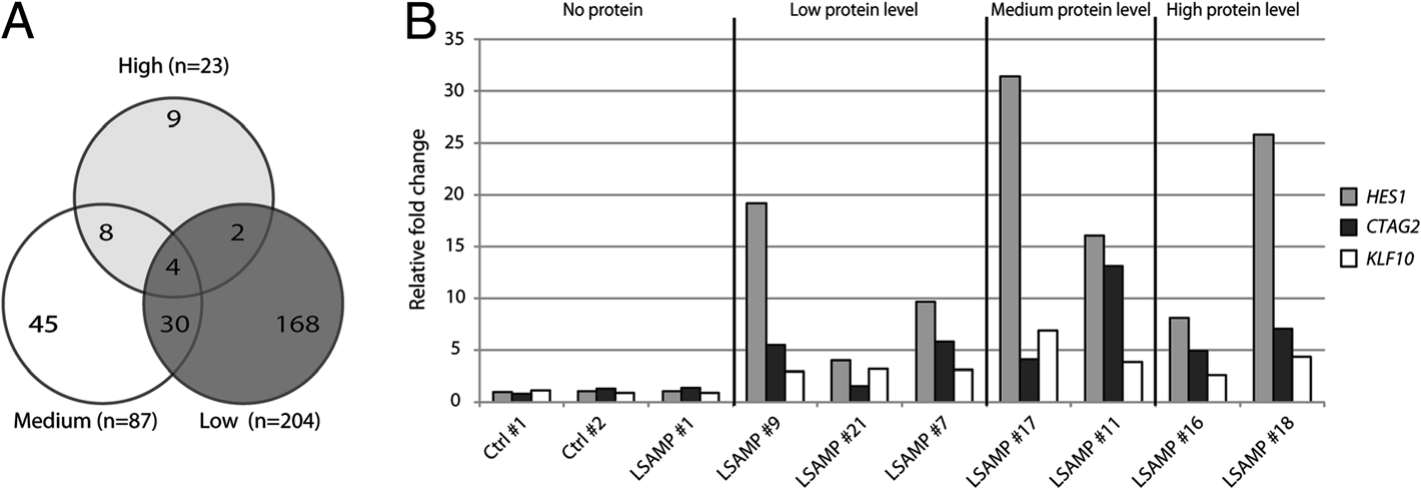
**Differential gene expression: A**) Venn diagram showing the number of differentially expressed genes in the clones with low (LSAMP #7, #9 and #21), medium (LSAMP #11 and #17) and high (LSAMP #16 and #18) LSAMP protein level compared to the average of the two control clones (Ctrl #1 and #2). **B**: Validation of the mRNA expression profiling results for the genes *HES1, CTAG2* and *KLF10,* determined by qRT-PCR. Fold change is compared to the average of the two control clones (Ctrl #1 and #2).

To identify possible mechanisms involved in tumor suppression by LSAMP, changes in gene expression in response to *LSAMP* reexpression were investigated by mRNA expression profiling. Seven clones with different levels of the LSAMP protein (LSAMP #7, #9 #11, #16, #17, #18 and #21) were compared to the two control clones (Ctrl #1 and #2). The analysis revealed that compared to the average expression of the two control clones, three genes, in addition to *LSAMP,* were differentially expressed in all seven clones (Figure 5A). These genes were hairy and enhancer of split 1 (*HES1)*, cancer/testis antigen 2 (*CTAG2)* and kruppel-like factor 10 (*KLF10)*, which were all overexpressed compared to the controls, and the upregulation was validated by qRT-PCR (Figure 5B). In addition, one clone (LSAMP #1) expressing *LSAMP* mRNA, but without detectable levels of the protein (Figure 2A), was included. Interestingly, this clone had similar levels to the two control clones, supporting that the presence of the LSAMP protein is the cause for the induction of these genes.

Of the 42 samples tested for LSAMP protein (Figure 2A), 30 had available mRNA expression profiling data ( [21] and Kresse *et al.,* unpublished). The expression levels of the genes *LSAMP, HES1*, *CTAG2* and *KLF10* were compared between samples with detectable (n = 7) and undetectable (n = 23) levels of the LSAMP protein. For *LSAMP*, the expression was higher in the samples with LSAMP protein (Additional file 6: Figure S5 A, *p* = 0.026, Mann–Whitney test). For *HES1*, *CTAG2* and *KLF10,* no differences in the median expression levels between the two groups were observed (Additional file 6: Figure S5).

## Discussion

Osteosarcomas are cytogenetically complex malignancies, with a vast number of DNA copy number aberrations. A recurrent deletion within 3q13.31 has been identified [6,13,14,27,28]. This deletion has so far only been described in one other cancer type [20], indicating that it is highly specific to osteosarcomas. This specificity argues that the deletion is not due to a fragile site and strongly suggests involvement of this region in osteosarcoma development. Thus, the deleted region is likely to contain sequences preventing or retarding osteosarcoma development or progression.

The simplest interpretation would be that the region harbors one or more tumor suppressor genes. Concordantly, three genes located within the deleted region have been proposed to have tumor suppressive functions in osteosarcomas: *LSAMP*[6,12-14], *LSAMP-AS3*[13] and *LSAMP-AS4*[13]. However, our results showed either no or low expression levels of *LSAMP-AS3* and *LSAMP-AS4* in all the samples investigated, including the control samples. The control samples originated from osteoblastic cultures, bone, brain and smooth muscle. In addition, we included the Universal Human Reference RNA, which is a pool of RNA from ten different human cell lines representing various cancer types, excluding osteosarcoma. As neither *LSAMP-AS3* nor *LSAMP-AS4* were expressed in any of these control samples, it is reasonable to argue that the low or lack of expression in the osteosarcoma samples is not due to inactivation, but a normal state for both non-cancerous and cancerous cells. The other genes located within the deleted region (only annotated by ENSEMBL, supported by GENCODE/ENCODE) have been annotated only recently, and was thus not investigated in this study. However, stranded total RNA-sequencing data of 10 osteosarcoma cells lines and the non-tumorigenic cell line iMSC#3 (both undifferentiated and with osteogenic differentiation), showed no detectable expression corresponding to any of these genes (Meza-Zepeda *et al.,* unpublished). Even though we cannot exclude that other genes within the deleted region are important for the development of osteosarcomas, several lines of evidence indicate that *LSAMP* is a tumor suppressor gene and the target for the deletion in 3q13.31. DNA methylation of the promoter region has been described, indicating that *LSAMP* is epigenetically inactivated in cancer [6,17], and absent or low expression of *LSAMP* has been reported to be a frequent characteristic of osteosarcomas [6,12-14]. We have previously shown that expression of *LSAMP* is associated with poor survival in a larger panel of osteosarcoma patients [6], and it has also been shown in patients with epithelial ovarian cancer [15]. In this study, we saw a trend towards poorer survival in patients with loss of *LSAMP* copy number, although not statistically significant, possibly due to the limited sample size (n = 18). Overexpression of *LSAMP* was shown to inhibit proliferation in a renal cell carcinoma cell line [17], whereas depletion of *LSAMP* promoted cell proliferation in osteoblasts [13]. These observations are consistent with the function of *LSAMP* to be a tumor suppressor gene. Our results add to these evidences as we showed reduced proliferation rate *in vitro* and inhibited tumor growth *in vivo* when the expression of *LSAMP* was restored.

By western blotting, we investigated whether the *LSAMP* transcript was translated, which to our knowledge has not been done in tumor samples. However, we did not find a clear correlation between the mRNA level of *LSAMP* and the protein level in our samples. Interestingly, one study has shown that in osteosarcomas with high mRNA levels of *LSAMP,* a premature termination codon was detected [14]. It is tempting to speculate that this is the cause for the apparent lack of translation in some of the samples. However, the premature termination codon was not found in any of 19 osteosarcoma cell lines based on available RNA-sequencing data (Meza-Zepeda *et al*., unpublished), indicating that this is not a frequent event of regulation in osteosarcomas. Taking into account that 11/23 clones transfected with *LSAMP* ORF failed to translate the transcripts to detectable levels, there could also be post-transcriptional regulation of *LSAMP*, even though these transcripts are lacking features of the endogenous mRNA. Also, as only the clones with detectable levels of the LSAMP protein had upregulated *HES1, CTAG2* and *KLF10,* our results indicate that the LSAMP protein is indirectly affecting the upregulation of these genes. However, it is also possible that the cells upregulated one or more of these genes to be able to grow in the presence of the *LSAMP* protein. On the other hand, there were no differential expression of these genes between the investigated samples with (n = 7) and without (n = 23) detectable levels of the LSAMP protein, based on mRNA expression profiling data ( [21], Kresse *et al.,* unpublished). This discrepancy could be explained by the relatively small size of the cohort with LSAMP protein. However, *LSAMP* is one of four IgLONs [29], which are cell adhesion molecules that function as dimers, referred to as Diglons [30]. LSAMP has been shown to only function as heterodimers with either Opioid-binding cell adhesion molecule (OBCAM) or Neurotrimin (NTM) [30]. If the dimerization partner of LSAMP is not present, it would most likely not function properly. *OBCAM* was not expressed in any of the investigated samples (n = 7 with LSAMP protein, n = 23 without LSAMP protein), but *NTM* was expressed at different levels. Furthermore, the non-transfected parental cell line IOR/OS14 had among the highest expression levels of *NTM* and ranking as number 11 out of the 30 samples (data not shown). Also, the majority of the cohort with LSAMP protein had low expression of *NTM*. Thus, it is possible that we do not find an upregulation of *HES1*, *CTAG2* and/or *KLF10* in these samples due to the lack of expression of a LSAMP dimerization partner.

*HES1, CTAG2* and *KLF10* have all been shown to play a role in cancer biology. *HES1* has been suggested to have both oncogenic and tumor suppressive functions [31,32], whereas *KLF10* has been suggested to be a tumor suppressor gene [33,34]. *CTAG2* is mainly expressed in the reproductive organs, in addition to a variety of cancers [35-37]. To our knowledge, the function of *CTAG2* is not known, but both *HES1* and *KLF10* have been shown to be involved in bone biology. HES1 has been shown to bind to bone-specific promoters together with the runt-related transcription factor 2 (RUNX2) and the retinoblastoma protein (RB) [38]. *KLF10* has been shown to have an impact on the proliferation of osteoblasts, osteoclasts and osteosarcoma cells [39,40], which could be the cause for the observed reduced proliferation rate. In a recent study, *LSAMP* clustered together with pro-apoptotic genes when gene expression changes of osteosarcoma cell lines were analyzed after induction of apoptosis [41]. Furthermore, depletion of *LSAMP* in osteoblasts had an effect on the expression of the proapoptotic genes *BCL2* and *BimEL*[13]. Although apoptosis could be a possible mechanism for *LSAMP* to suppress or delay tumors formation, we did not find evidence of apoptosis in our clones with *LSAMP-*reexpression*.* This is consistent with another study where *LSAMP* was overexpressed in a clear cell renal cell carcinoma cell line without any evidence of apoptotic cells [17].

The high frequency of the deletion in 3q13.31 and the specificity for osteosarcomas strongly suggest a functional role for this region and that it harbors a tumor suppressor gene important for the development of osteosarcomas. Our results indicate that among the genes investigated in 3q13.31, *LSAMP* is the target for the deletion. The function of *HES1* and *KLF10* in bone biology also implies a function for these genes in osteosarcoma development. Furthermore, the reduced proliferation rate *in vitro* and inhibited tumor growth *in vivo* is further pointing to a tumor suppressor function of *LSAMP*.

## Conclusions

We have identified a frequent deletion in osteosarcomas and shown *LSAMP* to be the target gene within the deletion. We believe that *LSAMP* is a tumor suppressor gene in osteosarcomas and that *LSAMP* suppress tumors by reducing the proliferation rate of cancer cells, possibly through upregulation of one or more of the genes *HES1, CTAG2* and *KLF10*.

## Materials and methods

### Samples

Clinical data for all osteosarcoma and control samples are listed in Additional file 1: Table S1.

Human osteosarcoma clinical samples (n = 39) were analyzed, of which 13 were primary or metastatic samples collected at the Norwegian Radium Hospital, Oslo, Norway and 26 were grown subcutaneously in immunodeficient mice as xenografts (suffix x), obtained either from the Norwegian Radium Hospital (n = 15) or the Department of Pathology, University of Valencia, Spain (n = 11). All tumors were diagnosed according to the current World Health Organization classification [42]. The informed consent used and the collection of samples were approved by the Ethical Committee of Southern Norway, Project S-06133 or the Institutional Ethical Committee of the University of Valencia. The samples were collected immediately after surgery, snap frozen in liquid nitrogen and stored at -80°C. The establishment and passing of xenografts were in accordance with national and institutional animal care guidelines.

In addition, osteosarcoma cell lines (n = 21) were analyzed. These were: G-292, 143B, HOS, MNNG/HOS, MG-63, OSA (SJSA-1), Saos-2, U-2 OS (n = 9) (American Type Culture Collection; ATCC), HAL, KPD, MHM, OHS (n = 4) (The Norwegian Radium Hospital), IOR/OS9, IOR/OS10, IOR/OS14, IOR/OS15, IOR/OS18, IOR/MOS, IOR/SARG (n = 7) (Istituto Ortopedico Rizzoli, Bologna, Italy), ZK-58 (Heinrich-Heine University, Düsseldorf, Germany) and CAL 72 (University College London, London, UK). The cells were grown in RPMI 1640 or DMEM (both Lonza) supplemented with 10% FBS (Fisher Scientific) and GlutaMAX (Life Technologies), at 37°C in a humidified atmosphere with 5% CO_2_. All cell lines were tested for mycoplasma and found negative, and authenticated as previously described [43].

Control samples (n = 15) were also analyzed. Normal long bone samples were purchased from Capital Biosciences (n = 2) or obtained from amputations of cancer patients at the Norwegian Radium Hospital (n = 4) or University College London (n = 1). The normal bone was collected as distant as possible from the tumor site, and SNP array confirmed normal DNA copy number. Primary osteoblast cultures (n = 2) isolated from human calvaria of different donors were purchased from ScienCell Research Laboratories. The non-tumorigenic cell lines (n = 3) HEPM, hFOB (both ATCC) and iMSC#3 were included; the latter being an immortalized human bone marrow-derived mesenchymal stromal cell line established in our laboratory (Skårn *et al*., unpublished). Commercial normal RNA samples (n = 3) were used, one from brain tissue (Life Technologies), one from smooth muscle (Clonetech Laboratories) and Universal Human Reference RNA (Agilent Technologies).

### DNA copy number and expression level

DNA copy number was determined either by high-resolution aCGH or the real-time PCR based assay TaqMan DNA Copy Number Assay (Life Technologies), as previously described [21]. aCGH was performed using the Affymetrix Genome-Wide Human SNP Array 6.0 (Affymetrix) and DNA copy number analysis was performed using the Nexus software (BioDiscovery), as previously described [21]. Expression level was determined using TaqMan Gene Expression Assays (Life Technologies) as previously described [6], and is hereafter referred to as qRT-PCR (quantitative real-time reverse transcription PCR). The expression level of the clinical samples and cell lines was analyzed as previously described [6], and the expression level of the clones was analyzed using the 2^-∆∆Ct^-method [44], with TATA box binding protein (*TBP)* as an endogenous reference. The assays used and their respective ID number are listed in Additional file 7: Table S2.

### Western blotting

Total protein lysate was run on a 4-12% Bis-Tris NuPAGE precast gel (Life Technologies) and transferred onto a PVDF membrane (Millipore). The antibodies used and their respective conditions are listed in Additional file 8: Table S3. The proteins were visualized using SuperSignal West Duration Substrate (Thermo Scientific).

### Vector construction and transfection

The *LSAMP* expression vector was constructed using Gateway Technology (Life Technologies), recombining a vector containing *LSAMP* open reading frame (ORF) (ID number OCAAo5051A0349D, imaGenes) with pT-REx-DEST30 (Life Technologies)*.* The cell line IOR/OS14 was stably transfected with either the expression vector (named *LSAMP* ORF) or the backbone vector using Lipofectamine 2000 (Life Technologies). Selection was performed using 450 μg/ml Geneticin (Life Technologies) for 14 days, after which the concentration was reduced to 225 μg/ml.

### Immunofluorescence confocal microscopy

The anti-LSAMP antibody was a kind gift from Dr. Aurea F. Pimenta, Vanderbilt University, Nashville, USA. The cells were grown on coverslips and fixed in 10% formalin solution (Sigma-Aldrich), rinsed in PBS and blocked in 5% FBS in PBS for 30 min before incubation with the antibody in 1:100 dilution in 5% FBS for 1 h at RT. After incubation, the cells were washed 3 × 5 min in 5% FBS and incubated in a 1:200 dilution of anti-mouse-IgG/Cy3 (Jackson ImmunoResearch Laboratories) for 30 min at RT. The cells were then washed 3 × 5 min in 5% FBS and rinsed in dH_2_O. The nuclei were stained using ProLong Gold Antifade Reagent with DAPI (Life Technologies). The fluorescence was visualized using a Zeiss LSM 510 confocal microscope (Zeiss) and pictures were taken of thin single plane sections.

### Proliferation rate

Proliferation rate was measured using the CellTiter 96 AQueous One Solution Cell Proliferation Assay (MTS) (Promega). Cells stably transfected with *LSAMP* ORF or the backbone vector were seeded in quadruplicates in a 96-well plate with 10,000 cells per well in 100 μl medium. The cell viability was measured after 96 h.

### *In vivo* tumorigenicity

Animal experiments were performed according to protocols approved by the National Animal Research Authority in compliance with the European Convention of the Protection of Vertebrates Used for Scientific Purposes (approval ID 1499 and 3275, http://www.fdu.no/). The experiments were performed as previously described [45].

### Apoptosis and cell cycle distribution

Apoptosis and cell cycle distribution were investigated using flow cytometry. For apoptosis, APO-BRDU (TUNEL) assay (Life Technologies) was performed, according to the manufacturer’s instructions. For investigation of cell cycle distribution, 2*10^6^ cells were harvested and resuspended in 200 μl ice-cold PBS and added to 4 ml ice-cold ethanol and incubated on ice for 45 min. Then, 6 ml of ice-cold staining buffer (SB: 0.5% BSA in PBS) was added, and the cells were centrifuged at 300 × *g,* at 4°C for 5 min. The pellet was resuspended in 1 ml SB and the centrifugation repeated. The cells were resuspended in 300 μl SB containing 2 μg/ml Hoechst 33342 (Sigma-Aldrich). For both assays, the LSR II UV Laser (BD Bioscience) was used, and the data was analyzed using FlowJo v8.8.7 software (Tree Star).

### Migration rate

The migration assay was performed using the IncuCyte system (Essen Bioscience).

### Differentiation

Adipogenic differentiation and Oil red O staining were performed as previously described [46], except that cells were seeded at a density of 3,000 cells/cm^2^.

For the osteogenic differentiation, the cells were seeded at a density of 4,500 cells/cm^2^. Osteogenesis was initiated with osteogenic induction medium containing 10 nM Dexamethasone, 3.5 mM β-Glycerolphosphate and 66.7 μM Ascorbic acid 2-phosphate (all from Sigma-Aldrich). The osteogenic induction medium was replaced every third day. To estimate the degree of differentiation, the wells were washed with PBS, fixed in ice-cold 70% ethanol for 1 h at 4°C, washed with ddH_2_O and subsequently stained with 0.4% Alizarin Red S solution (w/v, pH 4.2; Sigma-Aldrich) for 10 min at RT. The staining solution was removed by washing the cells 5 × in ddH_2_O, followed by a 15 min wash in PBS. The cells were dehydrated with 70% ethanol, followed by absolute ethanol and air-dried. This procedure was performed after 0, 14, 21 and 28 days of differentiation, respectively.

### mRNA expression profiling

RNA was isolated using the miRNeasy Mini Kit (QIAGEN GmbH). The RNA integrity was evaluated using the Agilent 2100 Bioanalyzer and the RNA nano 6000 kit (Agilent Technologies). For each sample, 500 ng of total RNA was used to make biotin-labeled and amplified cRNA with the Illumina TotalPrep Amplification Kit (Life Technologies). cRNA was hybridized to Illumina’s HumanHT-12 v4 Expression BeadChip as previously described [47]. Expression values were annotated using the file HumanHT-12_V4_O_R2_15002873_B.bgx (Illumina). The expression data was quantile normalized [48] in GenomeStudio Gene Expression module v1.9 (Illumina) and log_2_-transformed, and a rank product analysis [49] was performed in J-Express [50] using a q-value < 0.05 to identify significant changes of gene expression. The dataset has been deposited in the GEO data repository (http://www.ncbi.nlm.nih.gov/geo/, accession number GSE52089).

### Statistical analysis

Statistical analyses were performed using SPSS version 20. A p-value < 0.05 was regarded as statistically significant.

## Abbreviations

aCGH: array comparative genomic hybridization; CTAG2: Cancer/testis antigen 2, FBS, fetal bovine serum; HES1: Hairy and enhancer of split; KLF10: Kruppel-like factor 10; LSAMP: Limbic system-associated membrane protein; LSAMP-AS1: LSAMP antisense RNA 1; LSAMP-AS3: LSAMP antisense RNA 3; LSAMP-AS4: LSAMP antisense RNA 4; ORF: Open reading frame; PBS: Phosphate buffered saline; qRT-PCR: Quantitative real-time reverse transcription polymerase chain reaction; RT: Room temperature; SB: Staining buffer; TBP: TATA box binding protein.

## Competing interests

The authors declare that they have no competing interests.

## Authors’ contribution

TB carried out the experimental work, performed the statistical analysis and participated in data analysis and experimental design and wrote the manuscript. SHK participated in the statistical and data analysis, experimental design and writing of the manuscript. M. Skårn and M. Stabell participated in the experimental design and data analysis. RC performed the flow experiments and analysis. SL performed the immuofluorescence confocal microscopy and analysis. ALB provided biological material. OM and LAMZ conceived the study, participated in the design, data interpretation and manuscript writing. All authors read and approved the final manuscript.

## Supplementary Material

Additional file 1: Table S1 Clinical data for osteosarcoma samples and control samples. Click here for file

Additional file 2: Figure S1 Expression level of other genes in 3q13.31. The expression of *LSAMP-AS1*, *LSAMP-AS3* and *LSAMP-AS4* was investigated by qRT-PCR. The different expression levels are shown as relative percent to an endogenous reference gene (*TBP)* within the same sample*.* UHR: Universal Human Reference RNA, OB: Osteoblast. Click here for file

Additional file 3: Figure S2 Cell cycle distribution. A representative figure showing cell cycle distribution investigated by flow cytometry. **A** and **B**: Two clones with low levels of the LSAMP protein (**A**: #7 and **B**: #21), **C** and **D**: two control clones (**C**: #1 and **D**: #2) and **E**: non-transfected cells were included in the analysis. The experiment was performed twice. Click here for file

Additional file 4: Figure S3 Apoptosis. A representative figure showing apoptosis investigated by flow cytometry. Included in the analysis were **A**: Negative control cells, **B**: Positive control cells, **C-E**: Three clones with low levels of the LSAMP protein (**C**: #7, **D**: #9 and **E**: #21), **F** and **G**: two control clones (**F**: #1 and **G**: #2) and **H**: non-transfected cells. The experiment was performed thrice. Click here for file

Additional file 5: Figure S4 Migration rate. A representative figure showing the migration rate investigated by time-lapse photography using the IncuCyte. The migration of two clones with low levels of the LSAMP protein (#9 and #21), two control clones (#1 and #2) and non-transfected cells were monitored as relative wound density over time (h). The experiment was performed twice. Error bars represent standard deviations of the technical replicates (n = 6). Click here for file

Additional file 6: Figure S5 Expression levels of *LSAMP*, *HES1, CTAG2* and *KLF10* in samples with and without LSAMP protein. Shown are the expression levels of **A**: *LSAMP*, **B**: *HES1*, **C**: *CTAG2* and **D**: *KLF10* in samples with detectable (n = 7) and undetectable levels (n = 23) of the LSAMP protein. The expression level of *CTAG2* was detected by two probes in the bead array (probe ID ILMN 1787578 and ILMN 1715347), and shown in **C** is the median expression level of the two probes. The expression level of *KLF10* was detected by three probes (probe ID ILMN 1720080, ILMN 1659122 and ILMN 167594), and shown in **D** is the median expression level of the three probes. Click here for file

Additional file 7: Table S2 Overview TaqMan assays. Click here for file

Additional file 8: Table S3 Overview antibodies used for western blotting. Click here for file
