# Supplementary figures and images for: Reexpression of LSAMP inhibits tumor growth in a preclinical osteosarcoma model
Source: Mol Cancer. 2014 Apr 28;13:93. doi: 10.1186/1476-4598-13-93 (PMC4029956; doi:10.1186/1476-4598-13-93)

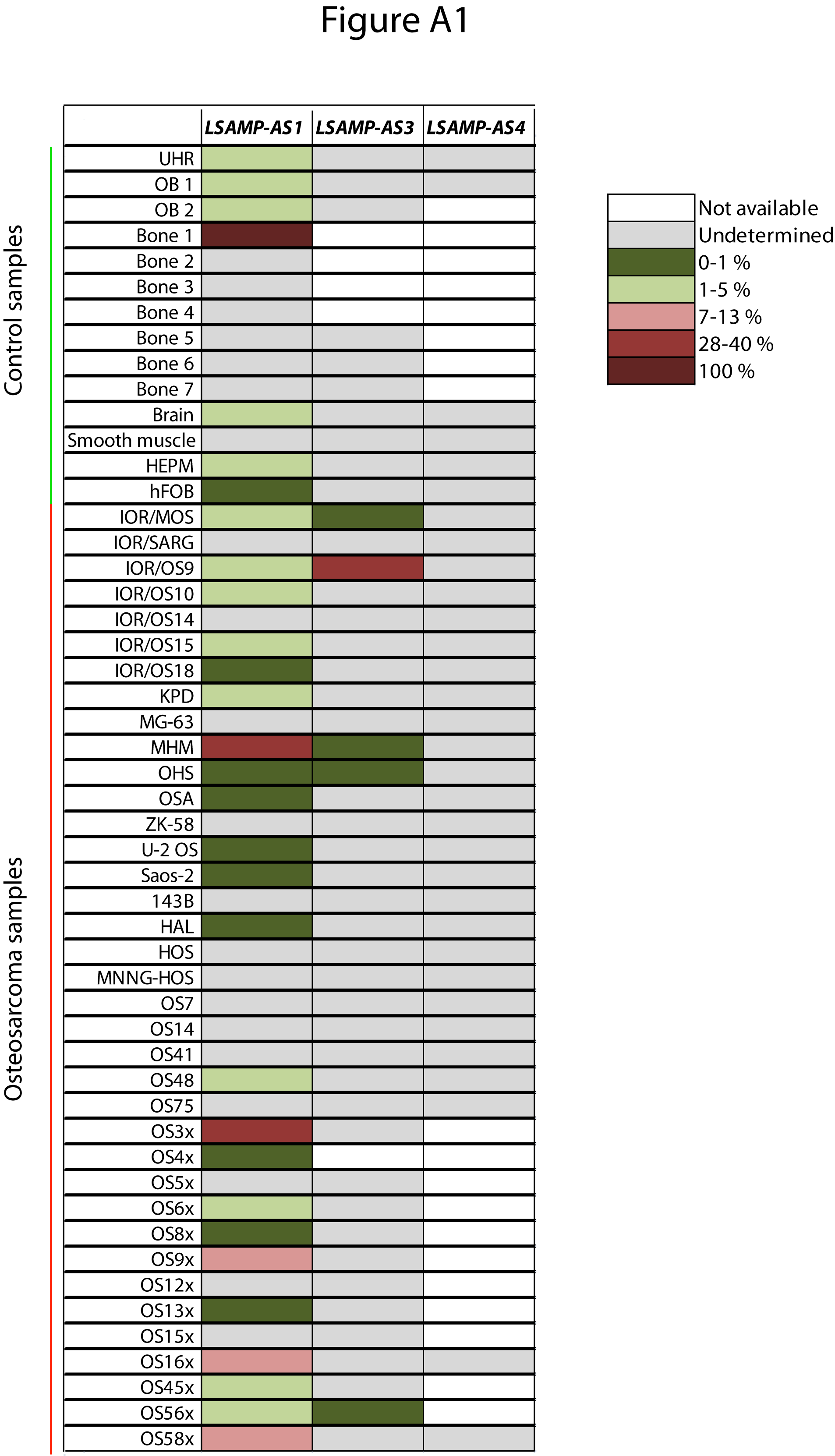

Supplement: Additional file 2: Figure S1 — Expression level of other genes in 3q13.31. The expression of LSAMP-AS1, LSAMP-AS3 and LSAMP-AS4 was investigated by qRT-PCR. The different expression levels are shown as relative percent to an endogenous reference gene (TBP) within the same sample. UHR: Universal Human Reference RNA, OB: Osteoblast. [file 1476-4598-13-93-S2.tiff]

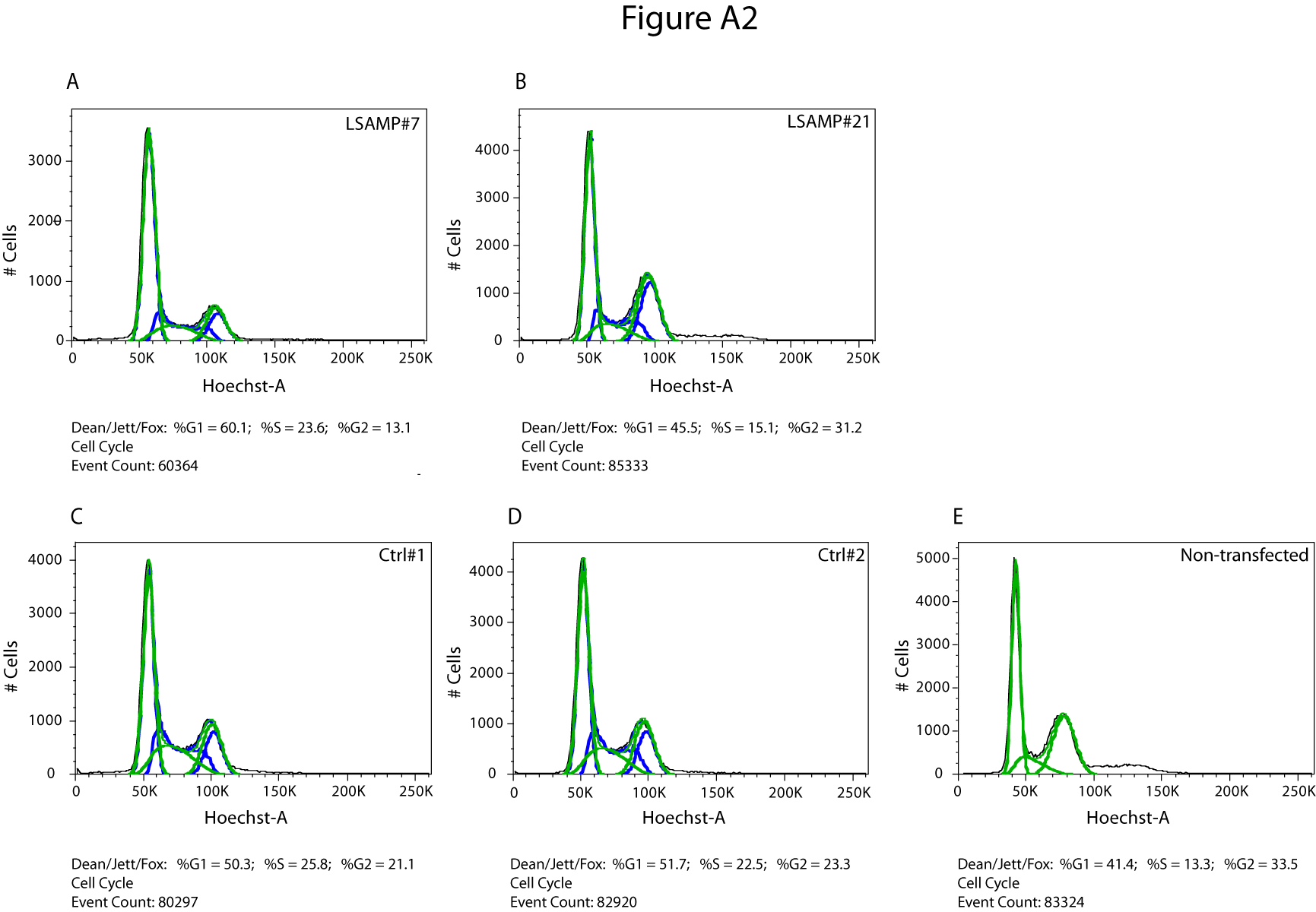

Supplement: Additional file 3: Figure S2 — Cell cycle distribution. A representative figure showing cell cycle distribution investigated by flow cytometry. A and B: Two clones with low levels of the LSAMP protein (A: #7 and B: #21), C and D: two control clones (C: #1 and D: #2) and E: non-transfected cells were included in the analysis. The experiment was performed twice. [file 1476-4598-13-93-S3.tiff]

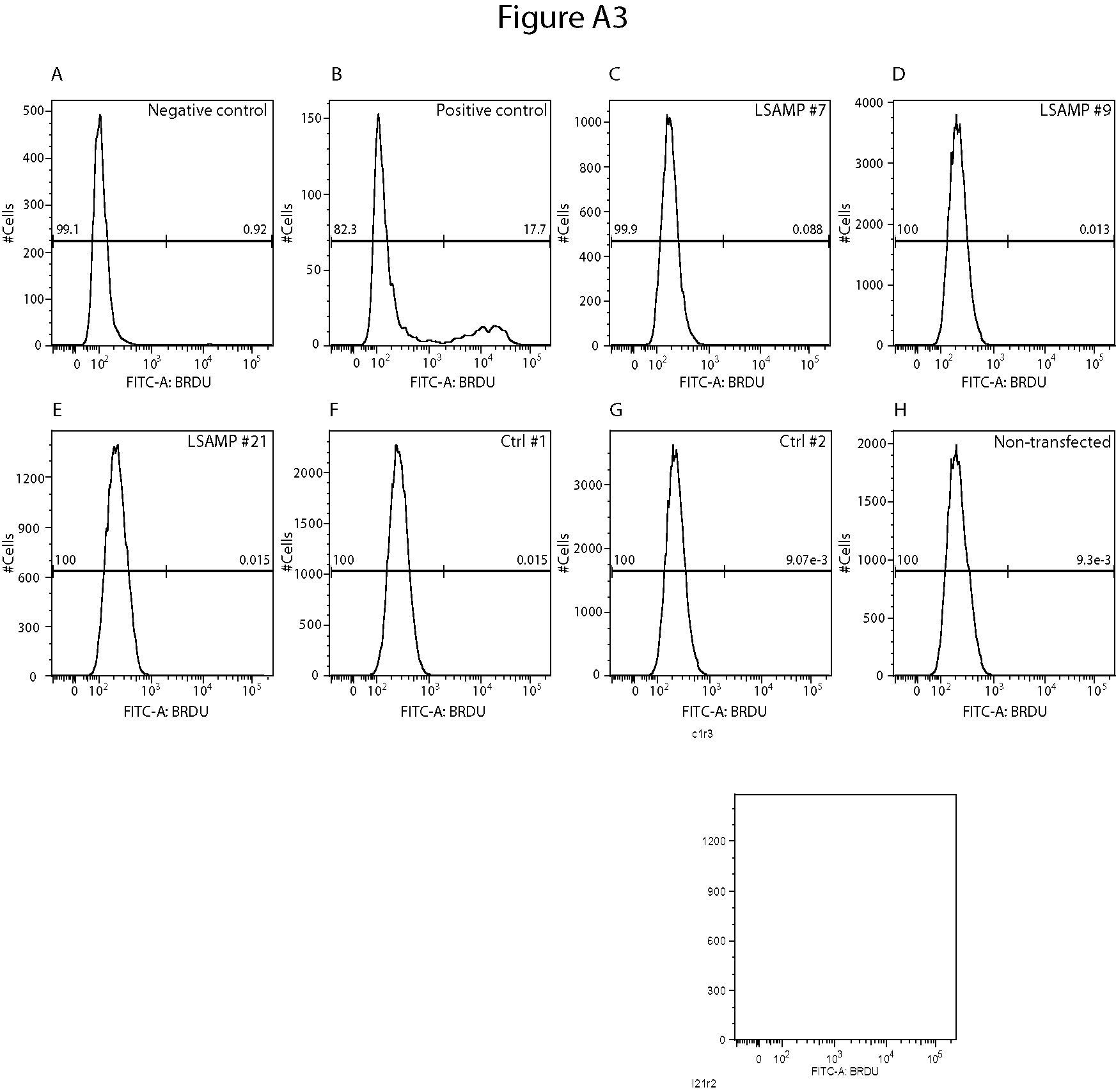

Supplement: Additional file 4: Figure S3 — Apoptosis. A representative figure showing apoptosis investigated by flow cytometry. Included in the analysis were A: Negative control cells, B: Positive control cells, C-E: Three clones with low levels of the LSAMP protein (C: #7, D: #9 and E: #21), F and G: two control clones (F: #1 and G: #2) and H: non-transfected cells. The experiment was performed thrice. [file 1476-4598-13-93-S4.tiff]

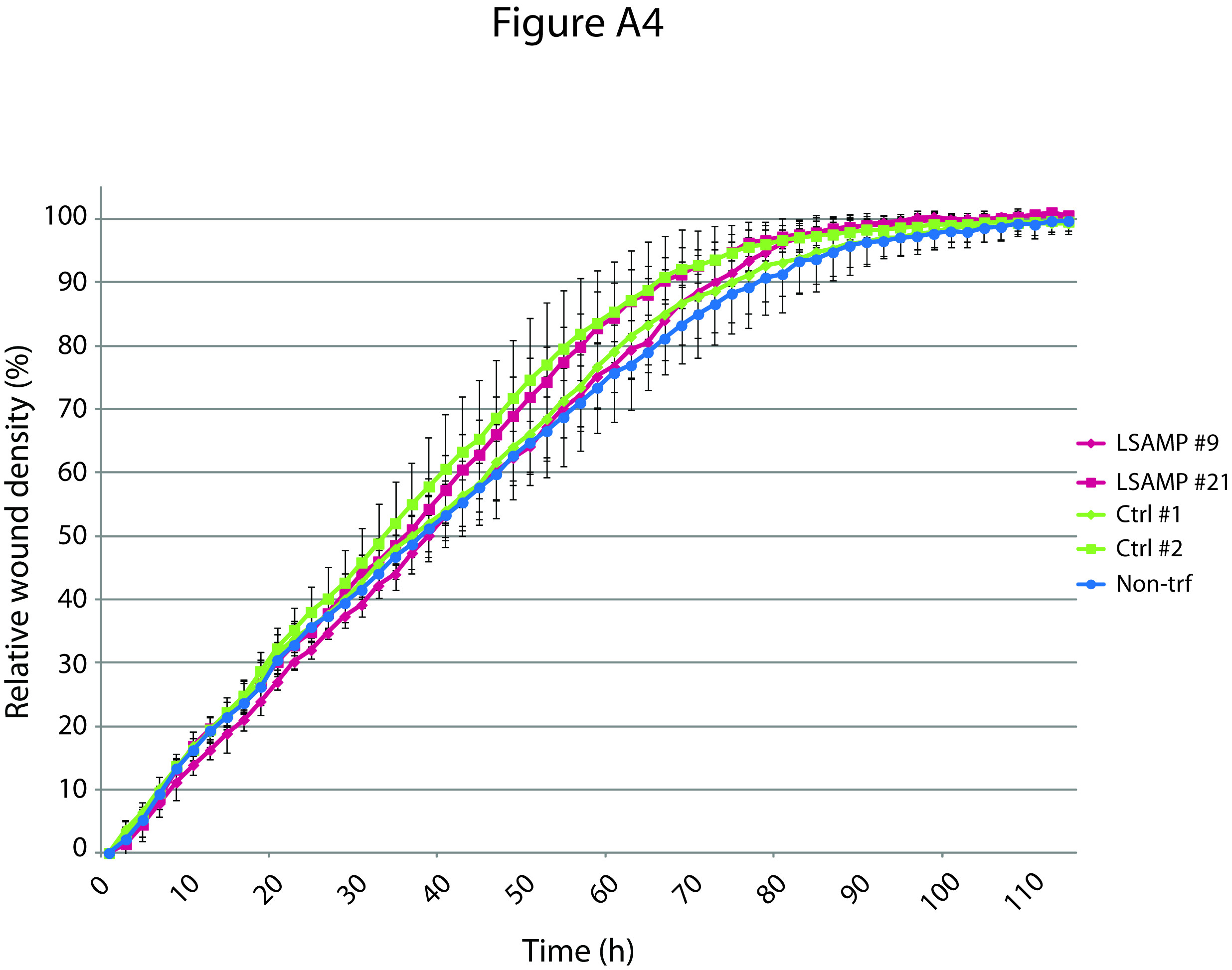

Supplement: Additional file 5: Figure S4 — Migration rate. A representative figure showing the migration rate investigated by time-lapse photography using the IncuCyte. The migration of two clones with low levels of the LSAMP protein (#9 and #21), two control clones (#1 and #2) and non-transfected cells were monitored as relative wound density over time (h). The experiment was performed twice. Error bars represent standard deviations of the technical replicates (n = 6). [file 1476-4598-13-93-S5.tiff]

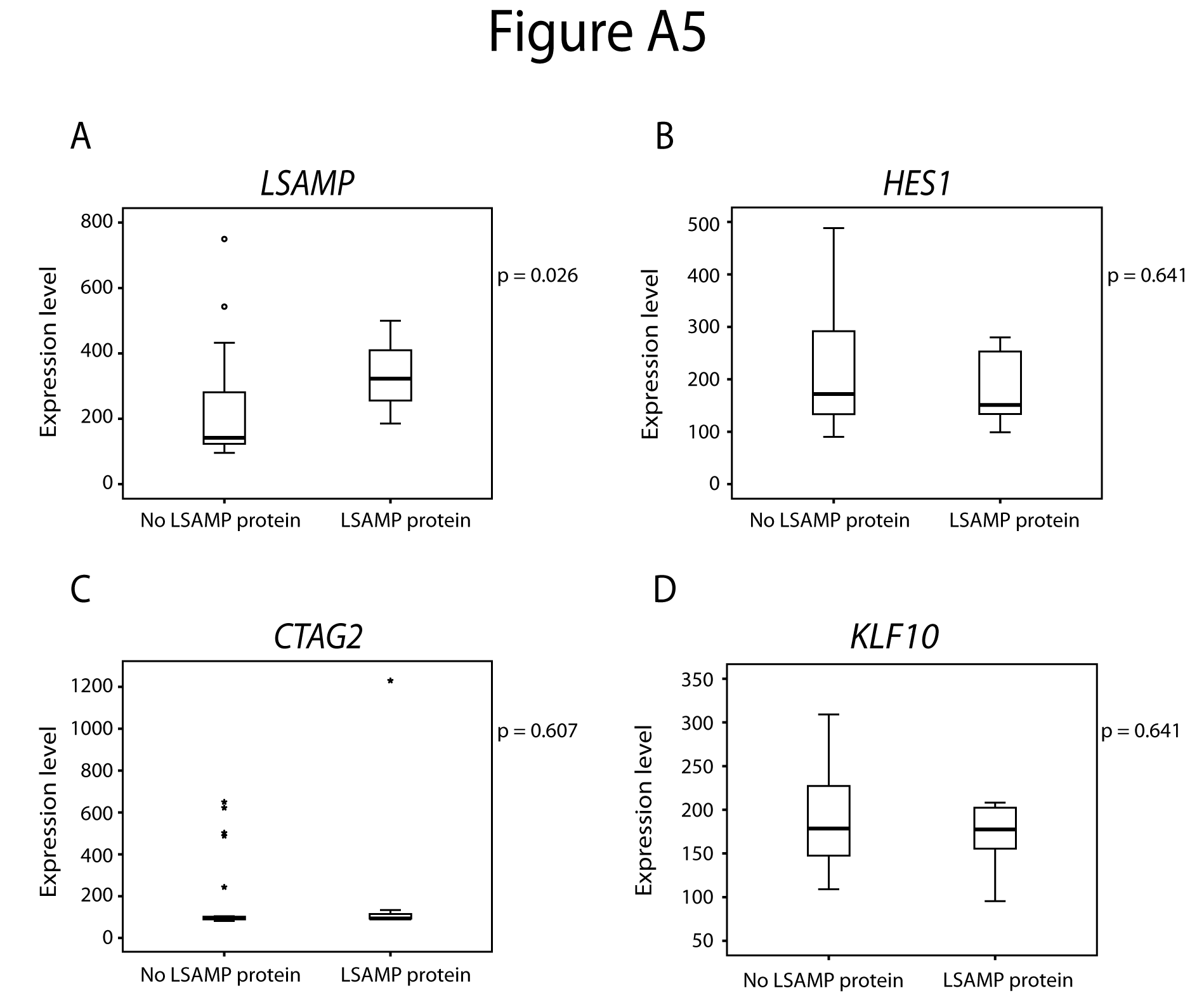

Supplement: Additional file 6: Figure S5 — Expression levels of LSAMP, HES1, CTAG2 and KLF10 in samples with and without LSAMP protein. Shown are the expression levels of A: LSAMP, B: HES1, C: CTAG2 and D: KLF10 in samples with detectable (n = 7) and undetectable levels (n = 23) of the LSAMP protein. The expression level of CTAG2 was detected by two probes in the bead array (probe ID ILMN 1787578 and ILMN 1715347), and shown in C is the median expression level of the two probes. The expression level of KLF10 was detected by three probes (probe ID ILMN 1720080, ILMN 1659122 and ILMN 167594), and shown in D is the median expression level of the three probes. [file 1476-4598-13-93-S6.tiff]
